# Bruceine D inhibits Cell Proliferation Through Downregulating LINC01667/MicroRNA-138-5p/Cyclin E1 Axis in Gastric Cancer

**DOI:** 10.3389/fphar.2020.584960

**Published:** 2020-11-24

**Authors:** Lin Li, Zhen Dong, Pengfei Shi, Li Tan, Jie Xu, Pan Huang, Zhongze Wang, Hongjuan Cui, Liqun Yang

**Affiliations:** ^1^ State Key Laboratory of Silkworm Genome Biology, Institute of Sericulture and Systems Biology, College of Sericulture and Textile and Biomass Science, Southwest University, Chongqing, China; ^2^ Department of Immunology, School of Basic Medicine, Southwest Medical University, Luzhou, China; ^3^ Cancer Center, Reproductive Medicine Center, Medical Research Institute, Southwest University, Chongqing, China; ^4^ Engineering Research Center for Cancer Biomedical and Translational Medicine, Southwest University, Chongqing, China; ^5^ Chongqing Engineering and Technology Research Center for Silk Biomaterials and Regenerative Medicine, Southwest University, Chongqing, China; ^6^ NHC Key Laboratory of Birth Defects and Reproductive Health (Chongqing Key Laboratory of Birth Defects and Reproductive Health, Chongqing Population and Family Planning Science and Technology Research Institute), Chongqing, China

**Keywords:** bruceine D, gastric cancer, LINC01667, microRNA-138-5p, cell proliferation

## Abstract

**Objective:** Gastric cancer is one of the most common malignant tumors. Bruceine D (BD) is one of the extracts of *Brucea javanica*. In recent years, it has been reported that BD has anti-tumor activity in some human cancers through different mechanisms. Here, this study try to explore the effect of BD on gastric cancer and its regulatory mechanism.

**Methods:** Cell proliferation ability was detected by 3-(4,5-dimethylthiazol-2-yl)-2,5-diphenyl tetrazolium bromide (MTT) assays, 5-bromo-2-deoxyuridine (BrdU) staining and soft agar colony formation assay, respectively. The tumor xenograft model was used to verify the effect of BD on the tumorigenicity of gastric cancer cells *in vivo*. Flow cytometry analysis and Western blot assay were performed to detect cell cycle and apoptosis. Gastric cancer cells were analyzed by transcriptome sequencing. The interaction between LINC01667, microRNA-138-5p (miR-138-5p) and Cyclin E1 was verified by dual luciferase experiment and RT-PCR assays.

**Results:** We found that BD significantly inhibited cell proliferation and induced cell cycle arrest at S phase in gastric cancer cells. Transcriptome analysis found that the expression of a long non-coding RNA, LINC01667, were significantly down-regulated after BD treatment. Mechanically, it was discovered that LINC01667 upregulated the expression of Cyclin E1 by sponging miR-138-5p. Furthermore, BD enhanced the chemosensitivity of gastric cancer cells to doxorubicin, a clinically used anti-cancer agent.

**Conclusion:** BD inhibit the growth of gastric cancer cells by downregulating the LINC01667/miR-138-5p/Cyclin E1 axis. In addition, BD enhances the chemosensitivity of gastric cancer cells to doxorubicin. This study indicates that BD may be used as a candidate drug for the treatment of patients with gastric cancer.

## Introduction

Gastric cancer is a malignant tumor originating from gastric mucosal epithelium. Despite the decline in the incidence and mortality of gastric cancer in the past 50 years, gastric cancer is still the fifth most commonly diagnosed cancer and the third largest cause of cancer-related death (Ferlay et al., 2010). In 2018, there are more than 1,033,701 new cases and 782,685 deaths of gastric cancer. On average, the incidence of gastric cancer in men is two to three times higher than that in women (Bray et al., 2018; Thrift and El-Serag, 2019). Although great progress has been made in a variety of treatments, including surgery, radiotherapy and chemotherapy, the prognosis of gastric cancer is still very poor (Lazar et al., 2016; Ilson, 2019). Therefore, screening or developing an effective drug is very important for the treatment of gastric cancer.

*Brucea javanica* (L.) Merr. is an herb which can play the similar role as artemisinin in anti-malaria and anti-cancer. *Brucea javanica* oil has been used in clinic, mainly for adjuvant treatment of digestive system tumors and lung cancer (Zhang et al., 2011; Wu et al., 2018; Zhu et al., 2018). The content of fatty oil in *Brucea javanica* seed is about 56.23%, including linoleic acid, oleic acid, etc. The chemical substances of *Brucea javanica* mainly include alkaloids, glycosides and flavonoids (Yu and Li, 1990). The seeds of *Brucea javanica* contain a variety of bitter ingredients similar to bitter lignin, including *Brucea javanica* bitter alcohol, bruceine A, B, C, D, E, F, G, H and so on. However, research on the bitter components of *Brucea javanica* is still in the primary initiation stage. At present, it has been confirmed that bruceine A has a certain inhibitory effect on the proliferation of non-small cell lung cancer cells, and induces apoptosis by causing DNA damage and activating mitochondrial apoptosis. In addition, bruceine A and D have antiparasite activity in goldfish (Wang et al., 2011). Bruceine D (BD) has anti-inflammatory activity and can be used as an effective leukocyte-endothelial cell adhesion inhibitor (Utoguchi et al., 1997).

BD is also one of these natural compounds extracted from *Brucea javanica* (Zhang et al., 1980; Shen et al., 2008). Previous studies have demonstrated that BD inhibits the proliferation of non-small cell lung cancer and induces apoptosis mediated by ROS mitochondria and JNK phosphorylation (Tan et al., 2019; Xie et al., 2019). BD inhibits the growth of hepatocellular carcinoma cells by targeting miR-95 (Xiao et al., 2014). Recent studies indicated that BD inhibits tumor growth and stem cell-like characteristics of osteosarcoma by inhibiting STAT3 (Wang et al., 2019). In chronic myeloid leukemia K562 cells, BD exerts its anti-tumor activity by inhibiting the phosphorylation of AKT and ERK and induces cellular apoptosis through mitochondrial pathway (Zhang et al., 2016). In addition, BD induces apoptosis in pancreatic cancer cells through inhibiting the anti-apoptotic activity of NF-κB, reducing mitochondrial membrane potential and activating redox sensitive p38-MAPK pathway (Lau et al., 2009; Yang et al., 2012).

However, the effect of BD in gastric cancer has not been explored. Based on previous findings, we hypothesize that BD may inhibit gastric cancer cell proliferation. This study firstly proves that BD has an anti-tumor activity in gastric cancer cells, which underpins a theoretical basis for the development and application of BD for the treatment of gastric cancer.

## Materials and Methods

### Cell Culture

The human gastric cancer cell lines HGC27, MKN45 and SGC7901, the human normal gastric epithelial cell line GES-1 and the human embryonic renal cell line 293FT were purchased from the American Type Culture Collection (ATCC, Rockville, MD, United States). Gastric cancer cell lines and GES-1 were cultured in Roswell Park Memorial Institute-1640 (RPMI-1640, Gibco) with 10% fetal bovine serum (FBS) and 1% penicillin-streptomycin (P/S, Invitrogen). 293FT cells were cultured in Dulbecco’s modified Eagle’s medium (DMEM, Gibco) containing 10% FBS, 1% P/S, 1% Geneticin 418 (G418, Invitgen), 1% non-essential amino acids (Invitgen), 1% sodium pyruvate (Invitgen) and 2% L-glutamine (Invitgen). 293FT transfection medium did not contain P/S or G418. All cells were cultured in a humidified incubator containing 5% CO_2_ at 37°C.

### Drug Treatment

BD was purchased from Chengdu Herbpurify (Chengdu, China) and then was dissolved in dimethyl sulfoxide (DMSO). The mother liquor concentration was 100 mM, and stored at −80°C. Human gastric cancer cells were treated with different concentrations of BD for different time. Cell morphology was photographed by an inverted microscope (Olympus, Japan).

### Cell Proliferation Assays

Cell proliferation was determined by 3-(4,5-dimethylthiazol-2-yl)-2,5-diphenyl tetrazolium bromide (MTT) assay. The cells were seeded to 96-well plates with 1,000 cells/well and cultured overnight in the incubator. The medium mixed with specified concentration of BD or DMSO. After a specific time, 20 μl MTT was added to each well and cells were cultured in the incubator for 2 h. The medium was sucked and 150 μl DMSO was added. The absorbance was measured at 560 nm using a microboard reader (Thermo Fisher, Waltham, MA, United States). The change of cell viability was calculated by formula (1-average absorbance/control group average absorbance) × 100%.

### 5-Bromo-2-Deoxyuridine Staining

The cells were seeded to a 24-well plate with 20,000 cells/well and cultured overnight in the incubator. Then they were cultured in the incubator for 48 h after the medium containing DMSO or BD was added to each well. After incubated with BrdU (Abcam, United States, 30 μg/ml) for 45 min, the cells were washed with phosphate buffered saline (PBS) and fixed in 4% paraformaldehyde (PFA) for 15 min. Subsequently, 200 μl 2 M hydrochloric acid was added to each well at 37°C for 20 min. After washing with PBS for three times, 10% goat serum containing 0.5% Triton X-100 (ZSGB-Bio, Beijing, China) was used for blocking at room temperature (RT) for 2 h. The cells were incubated with anti-BrdU monoclonal rat first antibody (1: 1,000) overnight at 4°C. The samples were incubated at RT for 2 h with Alexa FluorR^®^488 goat anti-mouse IgG second antibody (HG L; 1: 10,000, antioxidant). The nuclei were stained with DAPI (1: 1,000). The percentage of BrdU staining was calculated from at least five microscopic visual fields.

### Soft Agar Colony Formation Assay

The colony forming ability of gastric cancer cells was detected by soft agar colony formation assay (Hu et al., 2016). Briefly, 1 ml RPMI-1640 (Gibco) medium containing 0.6% agarose (Sigma-Aldrich, United States) was added to the 6-well plate as the basic agar. Then 1.5 ml RPMI-1640 (Gibco) medium containing 1,000 logarithmic cells, 0.3% agar mixed with a specific concentration of BD was added onto the basic agar. After cultured at 37°C for 2–3 weeks, the colonies were captured under microscope and counted after MTT staining.

### Flow Cytometry Analysis

Cells were treated with DMSO or BD for 48 h, and then collected for flow cytometry analysis. DMSO was used as control. Cell cycle and apoptosis analysis were performed as previously reported (Dong et al., 2017). For cell cycle assay, the cells were washed with cold PBS and then fixed with 75% ethanol at 4°C for 24 h. After washing with PBS, the cells were incubated in 200 μl PBS containing 1 μl 5 mg/ml propidium iodide (PI, BD, San Jose, CA, United States) and 2 μl 4 mg/ml RNaseA (Sigma Aldrich, Sigma Aldrich) at 37°C for 30 min. The cells were analyzed by a BD Accuri C6 flow cytometry (BD, United States). For the determination of cell apoptosis, the cells were incubated in 100 μl binding buffer containing 5 μl 50 μg/ml PI (BD, United States) and 5 μl Annexin-V (BD, San Jose, CA, United States) at room temperature for 20 min. The cells were analyzed by a BD Accuri C6 flow cytometry. All samples were analyzed by the FlowJo 7.6 software (BD, United States).

### Tumor Xenografts

Twelve 4-week-old female BALB/c nude mice (Beijing Laboratory Animal Research Center, China) were purchased and housed in the specific pathogen free room to acclimate for a week. The gastric cancer cells SGC7901 and MKN45 cells (1 × 10^6^) in 100 μl PBS were subcutaneously injected into both sides of each mouse. Seven days after cell injection, the mice were randomly divided into two groups. One group was intraperitoneally injected with BD (1.5 mg/kg), and the other group was injected with DMSO as control every two days for 14 days. The tumor growth was measured by caliper every day, and the tumor volume was calculated by formula (volume = length × width ^2^ × *π*/6). At the end of the experiment, the tumor was removed and weighed. All animal experiments were pre-approved by the Animal Ethics Committee of Southwest University. H&E and immunohistochemical (IHC) staining were performed as previous report (Yang L. et al., 2019).

### Western Blot Assay

Cells were collected and lyzed by using the RIPA lysis buffer (Beyotime, China) containing the complete protease inhibitor cocktail (Roche) and phosphatase inhibitors (Sigma Aldrich, St. Louis, MO, United States). Cell lysates were degenerated at 100°C for 15 min. Proteins were isolated with 8, 10 or 12% SDS-PAGE and transferred to the polyvinylidene fluoride (PVDF) membranes. The membrane was blocked with 5% bovine serum albumin (BSA) at RT for 2 h. The PVDF membrane was incubated with specific primary antibody against CDK2, Cyclin E1, Cyclin E2, PARP and Caspase3 (1: 1,000, cell Signal Technology, United States), *α*-tubulin (1: 1,000, Beyotime, China, United States) at 4°C overnight. The membrane was incubated with secondary antibodies (goat anti-mouse IgG and goat anti-rabbit IgG, 1:10,000, Beyotime, China) at RT for 2 h. The high sensitivity substrate of SuperSignal West Femto (Thermo Fisher, Waltham, MA, United States) was used to display protein bands, and Chemiscope 6000 chemiluminescence gel imaging system (Clinx Science, China) was used to capture proteins.

### RNA-Seq

MKN45 cells were treated with 1.2 μM BD or isometric DMSO for 48 h. Each group consisted of three biological repeats. Then the total RNA of each sample was extracted by using the RNAiso Plus (Total RNA extraction reagent) (TaKaRa, Dalian, China). Afterward, the samples were shipped with enough dry ice to Sangon Biotech Co., Ltd. (Shanghai, China) The quality and quantity of total RNA were confirmed by agarose gel electrophoresis. cDNA was prepared by using the cDNA Synthesis Kit (Illumina Inc., San Diego, CA, United States) and then was used for library construction and further Illumina deep sequencing. The tool package SOAP2 was used for alignments for short oligo nucleotide analysis, and only up to two mismatches with reference sequences were allowed. The quality of the original sequencing data was evaluated by FastQC. Through Trimmomatic for mass shearing, relatively accurate and effective data can be obtained. The ENSEMBL database (Homo_sapiens.GRCh37.55.cdna.all.fa) were used for the alignment of the sequenced reads to analyze the transcript levels. HISAT2 was used to compare the effective data of the samples to the reference genome and count the mapping information. RSeQC was used to perform redundant sequence analysis and insert fragment distribution analysis based on the comparison results. Qualimap was used to perform uniform distribution check and genome structure distribution analysis based on the comparison results. BEDTools were used to perform statistical analysis of gene coverage and analysis of sequence distribution on chromosomes. Heml 1.0: Heatmap Illustrator was used to draw a heatmap.

### Quantitative Real-Time PCR

After treated with DMSO or BD for 48 h, the cells were collected. Total RNA was extracted from cells using TRIzol^™^ reagent (Invitrogen) according to the manufacturer’s instructions. quantity real-time PCR (qRT-PCR) was conducted as previous report (Dong et al., 2020). Briefly, the total RNA was reversely transcribed to cDNA by using the M-MLV reverse transcriptase (Promega). SYBR PreMix Ex Taq II (Vazyme Biotech, Nanjing, China) was used to carry out qRT-PCR in 20 μL reaction system. RT-qPCR reaction was carried out by using a LightCycle96 real-time PCR system (Roche). The relative mRNA expression level was calculated by 2^−ΔΔCT^ method. The primers used in this study are listed in Table 1.

**TABLE 1:** Primers used in the quantity real-time PCR assay.

GAPDH-F	5′ AACGGATTTGGTCGTATTGGG3′
GAPDH-R	5′ CCTGGAAGATGGTGATGGGAT3′
LINC01002- F-1	5′TCCTAGCCTCCAGTTTTACCCT3′
LINC01002- R-1	5′CTAATAACCATCAACGTCTTCTGTG3′
LINC01667- F-1	5′ GATGACAGCAGTCGCAAAGG3′
LINC01667- R-1	5′ ATGACAGTGACCCAACCAACA3′
LINC01671- F-2	5′GTATCAGACGTGGGAAAGCAAT3′
LINC01671- R-2	5′CAGGAGCACATCAACAGGGA3′
LINC01001- F-1	5′CCCACTGATTCTACATTATGCTCC3′
LINC01001- R-1	5′TGCCGTGACGTAGGGTATGG3′
LINC00958- F-1	5′AAATTAGCCGGGCGTTGT3′
LINC00958- R-1	5′TGGAGTTTCGCTCTTGTTGC3′
LINC01278- F-1	5′GTGAGAACCTGGGGACGCTA3′
LINC01278- R-1	5′CGCCACGGTCTGAACTCTT3′
LINC00969- F-1	5′AAAGCAGATCCGTGGTTCCC3′
LINC00969- R-1	5′TCCGTCCCAAGACAGCAAA3′
LINC01089- F-1	5′CCAAGCCCAAGGACTCACA3′
LINC01089- R-1	5′CACGTTCTGCTCCTTCCACTT3′
CyclinE1-F-1	5′GTCCAAGTGGCCTACGTCAA3′
CyclinE1-R-1	5′AAGCAGCGAGGACACCATAA3′
U6-F	5′CTCGCTTCGGCAGCACA3′
U6-R	5′AACGCTTCACGAATTTGGGT3′
hsa-microRNA-138-5p	5′AGCTGGTGTTGTGAATCAGGCCG3′
MicroRNA unified reverse primer	5′TGGTGTCGTGGAGTCG3′

### Transfection and Infection

Human full-length LINC01667 RNA (NR_038377.1) and CCNE1 (NM_001238) were downloaded from the National Center for Biotechnology Information and ligated into a pCDH-CMV-MCS-EF1-GFP-puro vector by Changsha Youbao Biotechnology Co., Ltd. (Changsha, China). The packaging plasmid (pLP1, pLP2, pLP/VSVG) and overexpression plasmid were co-transfected into 293FT cell line by lipofectamine 2000 (Invitrogen, Carlsbad, CA, United States). At 48 h after transfection, the supernatant of the virus was collected and infected with gastric cancer cells by adding with polybrene. Drug resistant cell lines were selected with 2 mg/ml puromycin for subsequent experiments.

### Luciferase Reporter Assay

According to the predicted sites of TargetScanHuman 7.0 database (Agarwal et al., 2015), we constructed LINC01667 wild type and mutant double luciferase vectors (purchased from the Genecreate Biological Engineering Co., Ltd., Wuhan, China). 3 × 10^5^ cells were placed in each well and placed in a 24-well plate for cell transfection. 600 ng PRL-TK and 600 ng LINC01667-WT/MUT, 50 nM miRNA NC/microRNA-138-5p (miR-138-5p) mimics were co-transfected into 293FT and MKN45 cells in Opti-MEM serum-lowering medium (Life Technologies). After 8 h, 3 ml medium was refreshed in each well. After 48 h, luciferase reporter assay was carried out according to Promega production instructions. Luciferase activity was normalized to PRL-TK activity.

### Statistical Analysis

All experiments were confirmed by at least three technical and biological replicates. Quantitative data are expressed as the mean ± SD. Two-tailed Student’s t-test was performed for paired samples. *p* value < 0.05 was considered statistically significant.

## Results

### Bruceine D Inhibits Cell Growth and Proliferation in Gastric Cancer Cells

**FIGURE 1:**
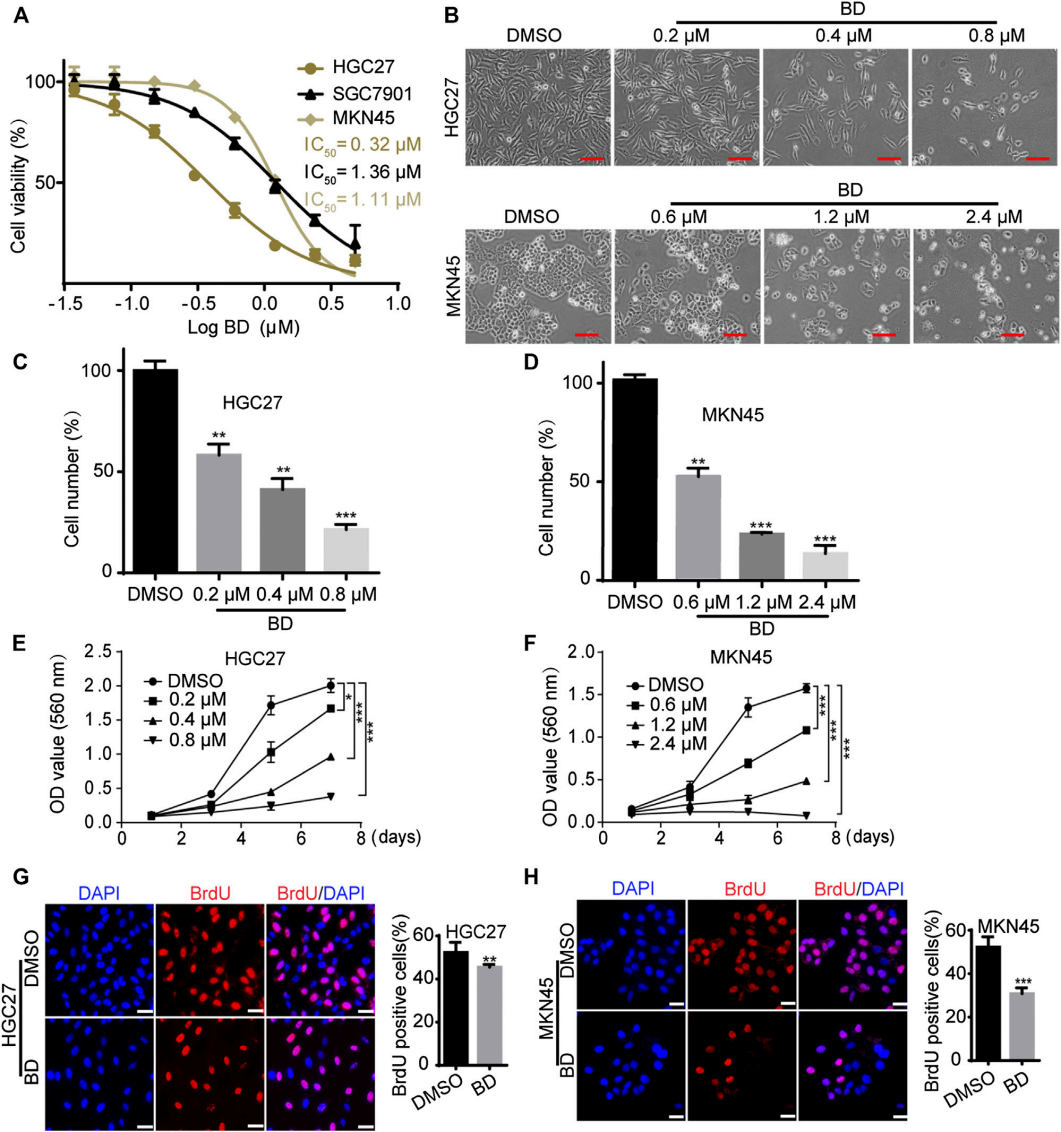
Bruceine D (BD) inhibits cell proliferation in gastric cancer cells. **(A)** HGC27, SGC7901 and MKN45 cells were treated with increasing concentrations of BD. After 48 h of BD treatment, cellular viability was determined by 3-(4,5-dimethylthiazol-2-yl)-2,5-diphenyl tetrazolium bromide (MTT) assay. Non-linear regression analysis was performed to determine IC_50_ values. **(B)** The morphology of HGC27 and MKN45 cells. HGC27 cells were treated with BD in different concentrations of 0.2, 0.4, and 0.8 μM for 48 h. MKN45 cells were treated with BD in different concentrations of 0.6, 1.2, and 2.4 μM for 48 h. Dimethyl sulfoxide (DMSO) was used as control. Scale bar = 50 μm. **(C,D)** Counting the number of cells treated as in **(B)**, and the histogram showed the quantity of cell proliferation rate. Cell numbers of DMSO-treated group were regarded as 100%. **(E,F)** Cell growth was monitored using MTT assays in cells treated with BD at the indicated times and concentrations. **(G,H)** Immunofluorescence staining for 5-bromo-2-deoxyuridine (BrdU) was performed. DAPI was used for nuclear staining. DMSO was used as control. Scale bar = 20 μm. The histogram shows the quantification of BrdU-positive HGC27 and MKN45 cells. All experiments were repeated at least three times. All data were used as mean ± SD, significant difference was tested by the two tailed and unpaired student’s t-test. Error bars, *p < 0.05, **p < 0.01, and ***p < 0.001.

To explore the biological function of BD in gastric cancer cells, the viability of cells treated with increasing doses of BD for 48 h was detected by MTT assay. The results showed that the IC_50_ of HGC27 and MKN45 cells were 0.32 and 1.11 μM, respectively (Figure 1A). According to the IC_50_ value, we selected the appropriate concentration of BD to treat gastric cancer cells (0.4 μM for HGC27 and 1.2 μM for MKN45). Observed by microscopy, HGC27 and MKN45 cells treated with BD showed significant morphological changes, and cell numbers decreased in a dose-dependent manner (Figures 1B–D). To confirm this result, MTT and BrdU staining assays were performed. MTT assay showed that gastric cancer cells treated with BD showed a sharp decline in the growth curve, compared to the DMSO group (Figures 1E,F). BrdU staining analysis also showed that the percentage of BrdU-positive cells treated with BD for 48 h was significantly lower than that in the DMSO group (Figures 1G,H). These results demonstrate that BD dramatically inhibits cell growth and proliferation in gastric cancer cells.

### Bruceine D Inhibits Cell Proliferation by Inducing Cell Cycle Arrest at the S Phase

**FIGURE 2:**
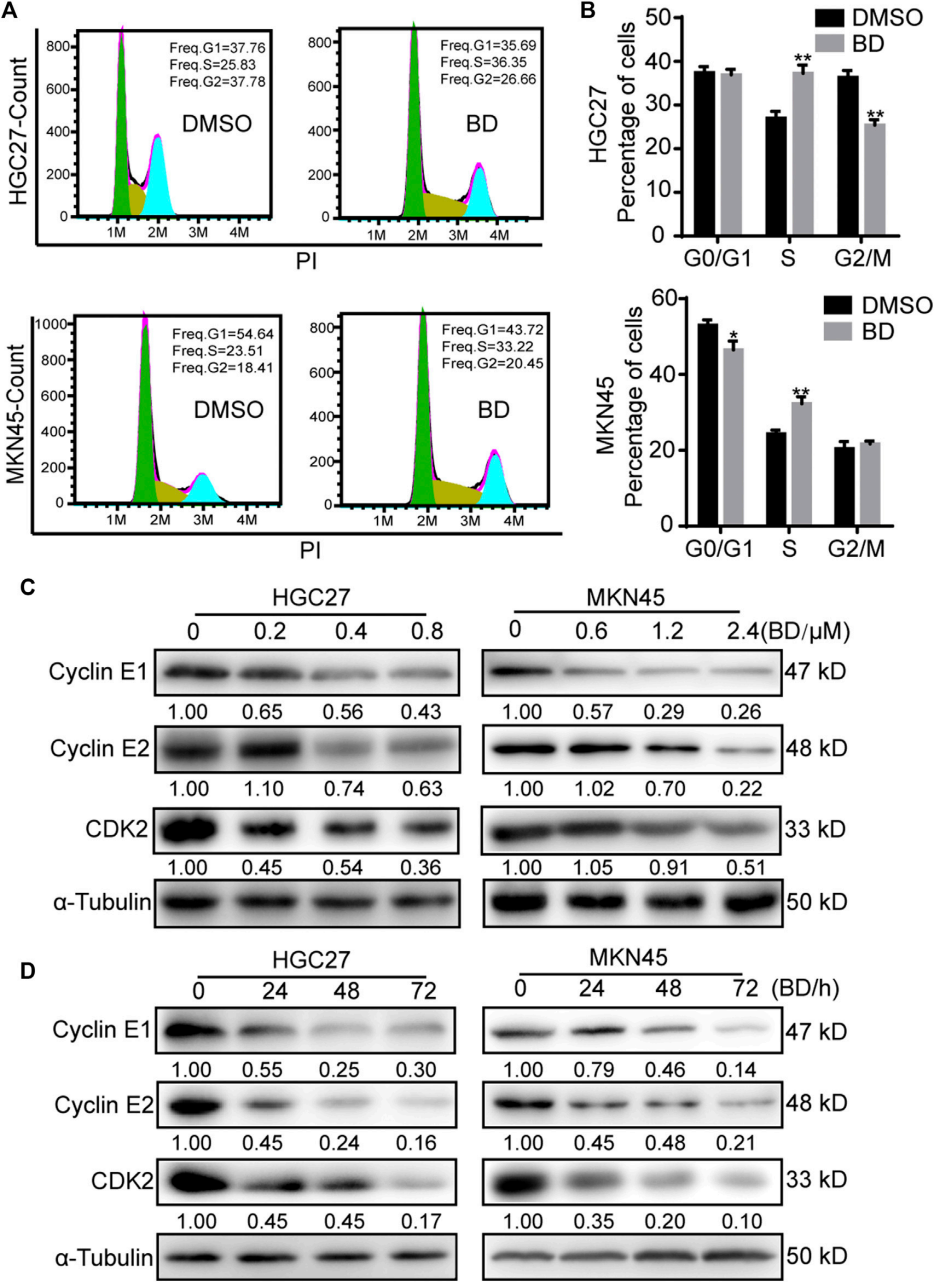
Bruceine D (BD) induces cell cycle arrest at S phase. **(A)** The cell cycle of gastric cancer cells were performed using flow cytometry. HGC27 and MKN45 cells were treated with BD at concentrations of 0.4 and 1.2 μM for 48 h, respectively. DMSO was used as control. **(B)** The histogram demonstrates the quantification of percentage of indicated HGC27 and MKN45 cells in different phase. **(C,D)** The expression of Cyclin E1, Cyclin E2 and CDK2 were determined using Western blot analysis after cells were treated with BD at different concentrations **(C)** or at different time points **(D)**. α-Tubulin was used as control. The gray ratio of Cyclin E1/α-Tubulin, Cyclin E2/α-Tubulin and CDK2/α-Tubulin was calculated. All experiments were repeated at least three times. All data were used as mean ± SD, significant difference was tested by the two tailed and unpaired student’s t-test. Error bars, *p < 0.05, **p < 0.01, and ***p < 0.001.

Cell proliferation is usually regulated by the procession of cell cycle. In order to understand the mechanism of BD in the inhibition of the proliferation of gastric cancer cells, we used flow cytometry to analyze the cell cycle of gastric cancer cells treated with BD for 48 h. The results showed that BD induced cell cycle arrest at the S phase (Figures 2A,B). To confirm this result, we detected the expression of CDK2, Cyclin E1 and Cyclin E2 that promoted cells to go through the G1 checkpoint by Western blot analysis. We found that the expression levels of CDK2, Cyclin E1 (CCNE1) and Cyclin E2 (CCNE2) were decreased in BD-treated cells in a dose- and time- dependent manner (Figures 2C,D). These results suggest that BD induces cell cycle arrest at the S phase in gastric cancer cells.

### Bruceine D Inhibits Clonogenicity and Tumorigenecity of Human Gastric Cancer Cells

**FIGURE 3:**
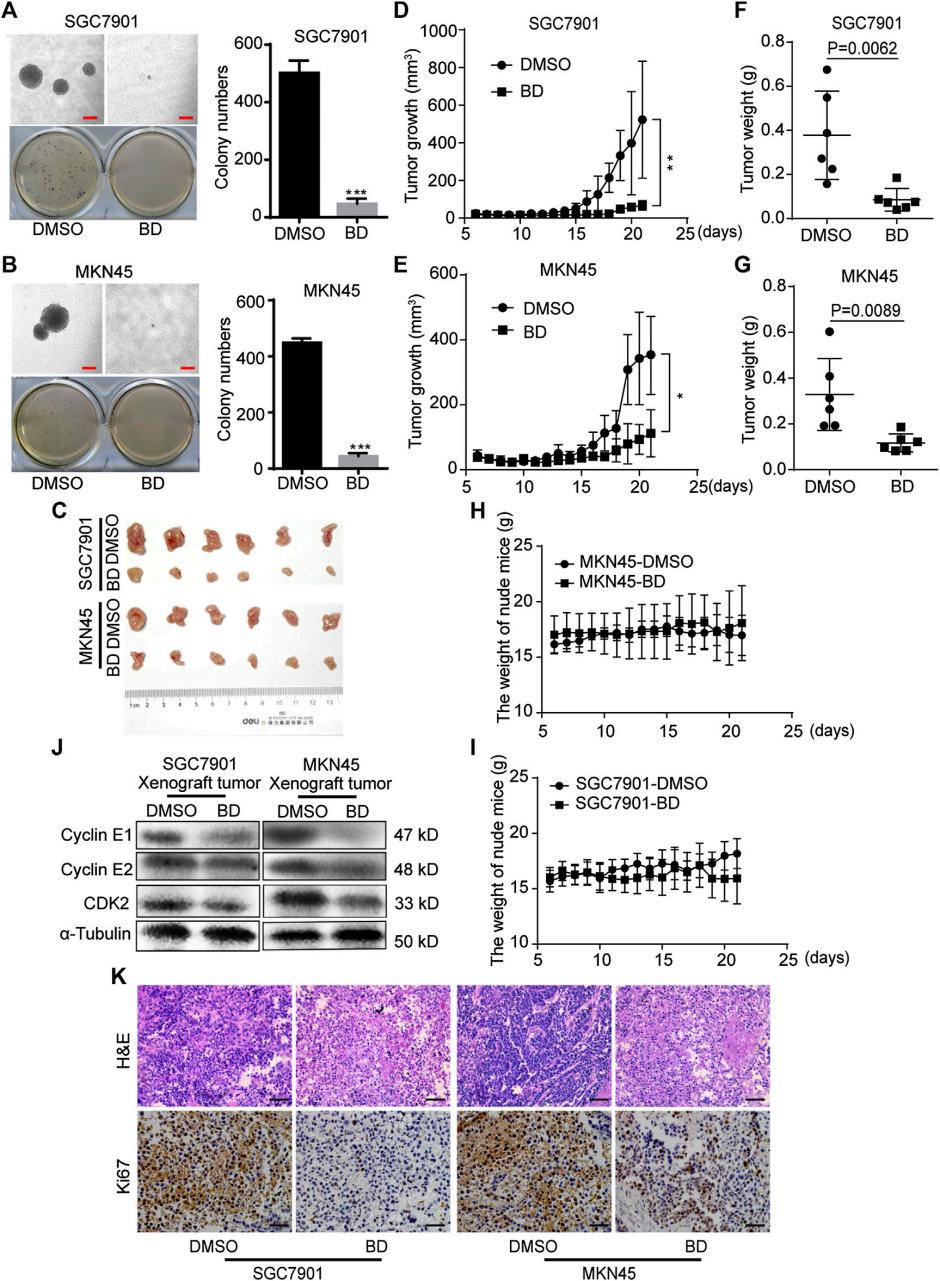
Bruceine D (BD) inhibits clonogenicity and tumorigenecity of human gastric cancer cells. **(A,B)** Soft agar assays were performed, and the results were quantitated in SGC7901 **(A)** and MKN45 **(B)** cells treated with 1.2 μM BD for two weeks. DMSO was used as control. Scale bar = 100 μm. **(C)** Image of xenograft tumors from mice. **(D,E)** Tumor growth curves in mice were calculated **(F,G)** Tumor weight removed from mice were measured. **(H,I)** The body weight of nude mice were measured. **(J)** The expression levels of CDK2, Cyclin E1 and Cyclin E2 were determined using Western blot assays in xenograft tumors. α-Tubulin was used as control. **(K)** H&E staining and immunohistochemical staining for Ki67 were performed in xenograft tumors. Scale bar = 50 μm. All data were used as mean ± SD, significant difference was tested by the two tailed and unpaired student’s t-test. Error bars, *p < 0.05, **p < 0.01, and ***p < 0.001.

In order to study the effect of BD on tumor growth in gastric cancer cells, soft agar colony formation assay *in vitro* and subcutaneously xenograft *in vivo* were carried out. In the soft agar colony formation assay, it was revealed that fewer and smaller cell colonies were produced in the BD-treated group, compared with the DMSO control group (Figures 3A,B). Next, we examined the effect of BD on tumor growth in the nude mice. The results showed that BD significantly inhibited tumor growth (Figure 3C), and the tumor weight in the BD-treated group was significantly lighter than those in the control group (Figures 3D–G). There was no significant change in body weight and behavior of mice treated with BD (Figures 3H,I). In addition, we detected cell cycle-related proteins expressed in the xenograft tumors obtained from mice by using the Western blot. The results showed that the expressions of these cell cycle-related proteins CDK2, Cyclin E1 and Cyclin E2 were significantly down-regulated after BD treatment (Figure 3J). H&E and IHC staining further supported the above results. H&E staining showed that the number of cells was decreased and the nucleus became smaller in BD-treated group. IHC staining showed that the expression of Ki67, a marker of cell proliferation, was significantly reduced in BD-treated group (Figure 3K). These results suggest that BD significantly inhibits the tumorigenecity of gastric cancer cells.

### Bruceine D Inhibits the Proliferation of Gastric Cancer Cells by Inhibiting LINC01667

**FIGURE 4:**
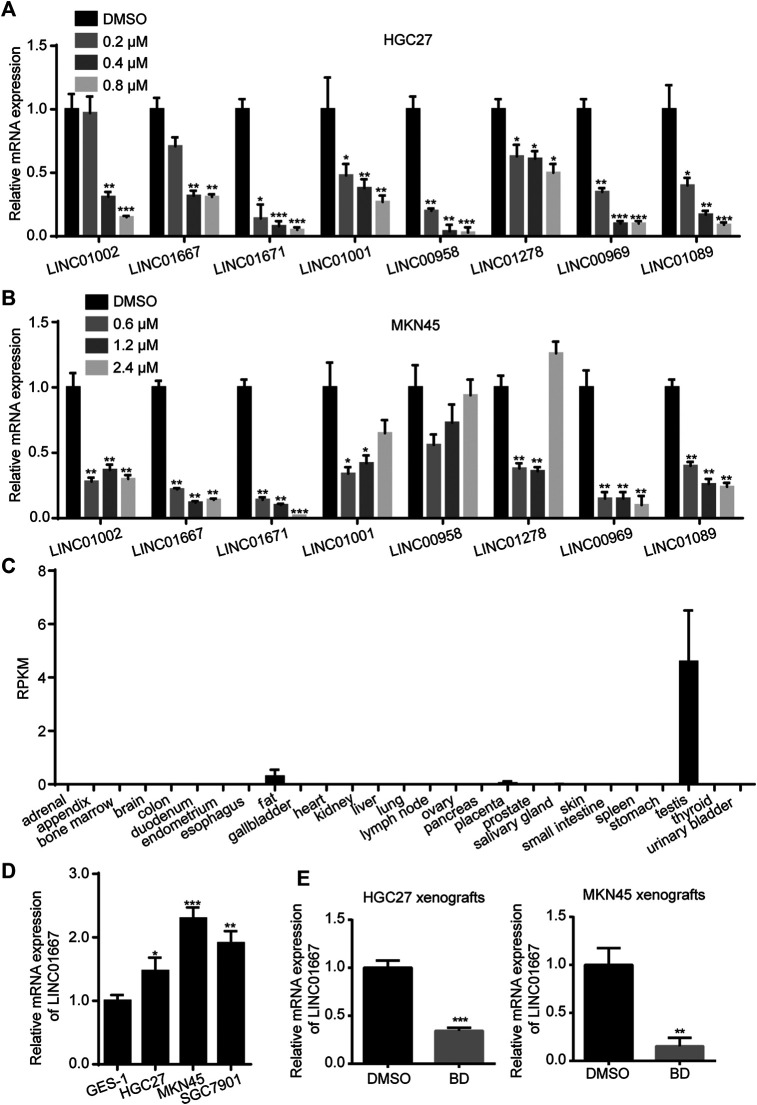
Bruceine D inhibits the expression of LINC01667. **(A,B)** The mRNA expression levels of several differential long non-coding RNAs weres detected by quantity real-time PCR (qRT-PCR) experiment. **(C)** The expression of LINC01667 in various parts of the human body. **(D)** The mRNA expression levels of LINC01667 in GES-1 and gastric cancer cell lines were detected by qRT-PCR experiment. **(E)** The mRNA expression levels of LINC01667 in HGC27 and MKN45 xenografts were detected by qRT-PCR experiment. All data were used as mean ± SD, significant difference was tested by the two tailed and unpaired student’s t-test. Error bars, *p < 0.05, **p < 0.01, and ***p < 0.001.

**FIGURE 5:**
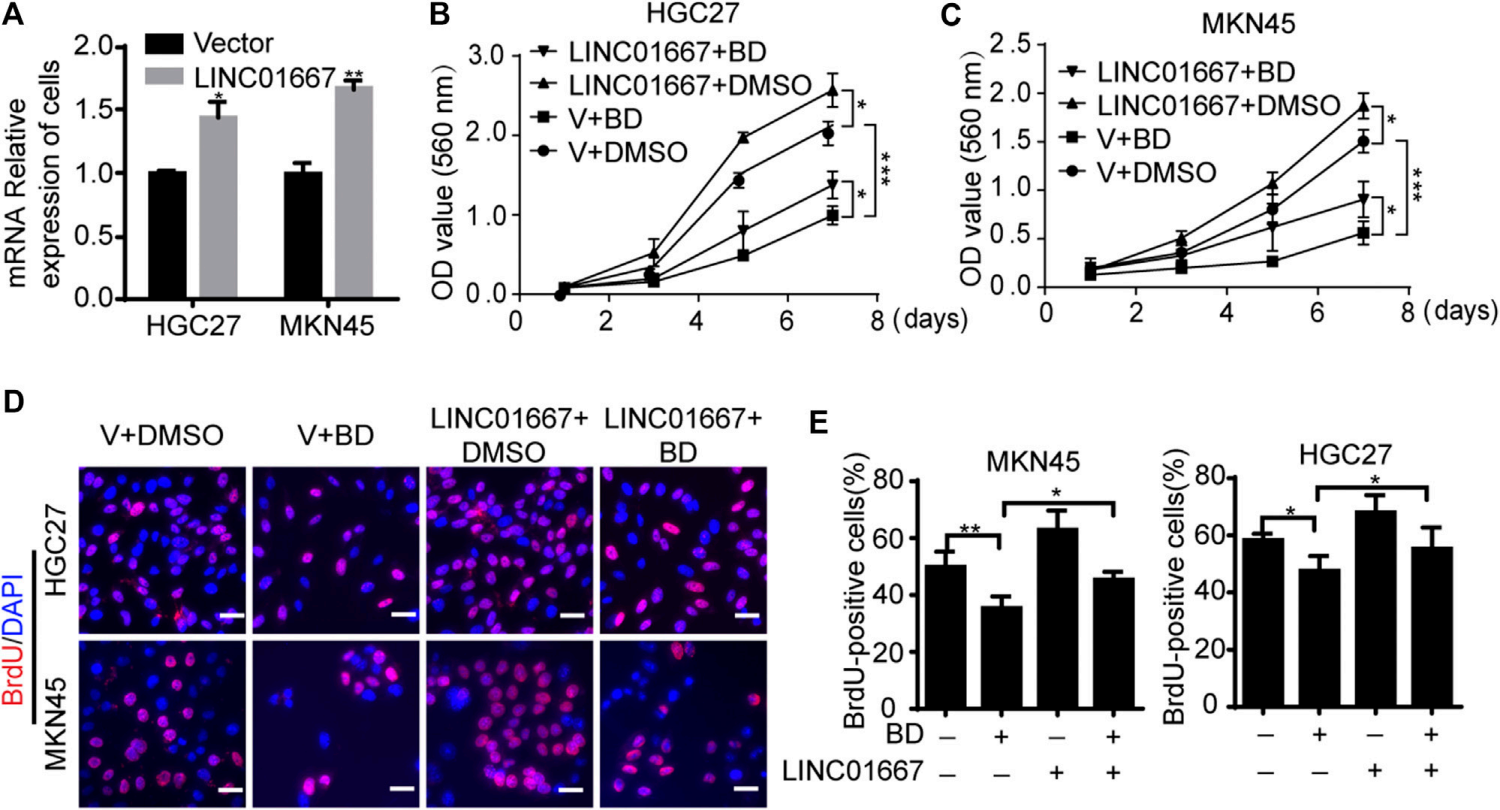
Bruceine D (BD) inhibits the proliferation of gastric cancer cells by inhibiting LINC01667. **(A)** The expression of LINC01667 in gastric cancer cell line was detected by quantity real-time PCR (qRT-PCR) assays. **(B,C)** Growth curve of LINC01667 overexpressing HGC27 and MKN45 cells after treating with BD at concentrations of 0.4 and 1.2 μM for the indicated time, respectively. Dimethyl sulfoxide (DMSO) and empty vector were used as control. **(D)** 5-bromo-2-deoxyuridine (BrdU)-positive cells in LINC01667-overexpressed HGC27 and MKN45 cells after treating with BD at concentrations of 0.4 and 1.2 μM for 48 h, respectively. DMSO and empty vector were used as control. Scale bar = 20 μm. **(E)** Quantification of BrdU-positive HGC27 and MKN45 cells in **C**. All data were used as mean ± SD, significant difference was tested by the two tailed and unpaired student’s t-test. Error bars, *p < 0.05, **p < 0.01, and ***p < 0.001.

In order to reveal how BD inhibits cell proliferation, a transcriptome analysis was performed on gastric cancer cells treated with BD or DMSO. In recent years, long non-coding RNA (lncRNAs) has been proved to play an important role in epigenetic regulation, cell cycle regulation and cell differentiation regulation (Geisler and Coller, 2013; Cech and Steitz, 2014). Besides, lncRNAs are emerging as a kind of new prognostic, diagnostic and therapeutic targets for cancer (Huarte, 2015), especially are important in gastric cancer (Wei et al., 2020). Through the analysis of the transcriptome data, we screened 89 lncRNA with significant changes in expression level (Supplementary Figure S1). Then we further screened out LINC01667, which may be a potential differential expressed lncRNA, through qRT-PCR analysis (Figures 4A,B) and database (https://www.ncbi.nlm.nih.gov/Gene/) (Figure 4C). LINC01667 was rarely expressed in gastric cell GES-1, but it was highly expressed in gastric cancer cell lines (Figure 4D). Importantly, LINC01667 mRNA expression was also significantlt decreased in both HGC27 and MKN45 xenografts treated by BD (Figure 4E). These data suggested that LINC01667 could play an oncogenic role in gastric cancer. In order to verify whether LINC01667 has an effect on the proliferation of gastric cancer cells, we constructed LINC01667 overexpression vector (Figure 5A). Then MTT (Figures 5B,C) and BrdU assays (Figures 5D,E) were carried out. The results showed that overexpression of LINC01667 could rescue the inhibition of cell activity induced by BD treatment. In summary, these results suggest that BD inhibits cell proliferation by inhibiting LINC01667.

### LINC01667 Sponges MicroRNA-138-5p and Upregulates Cyclin E1 Expression

**FIGURE 6:**
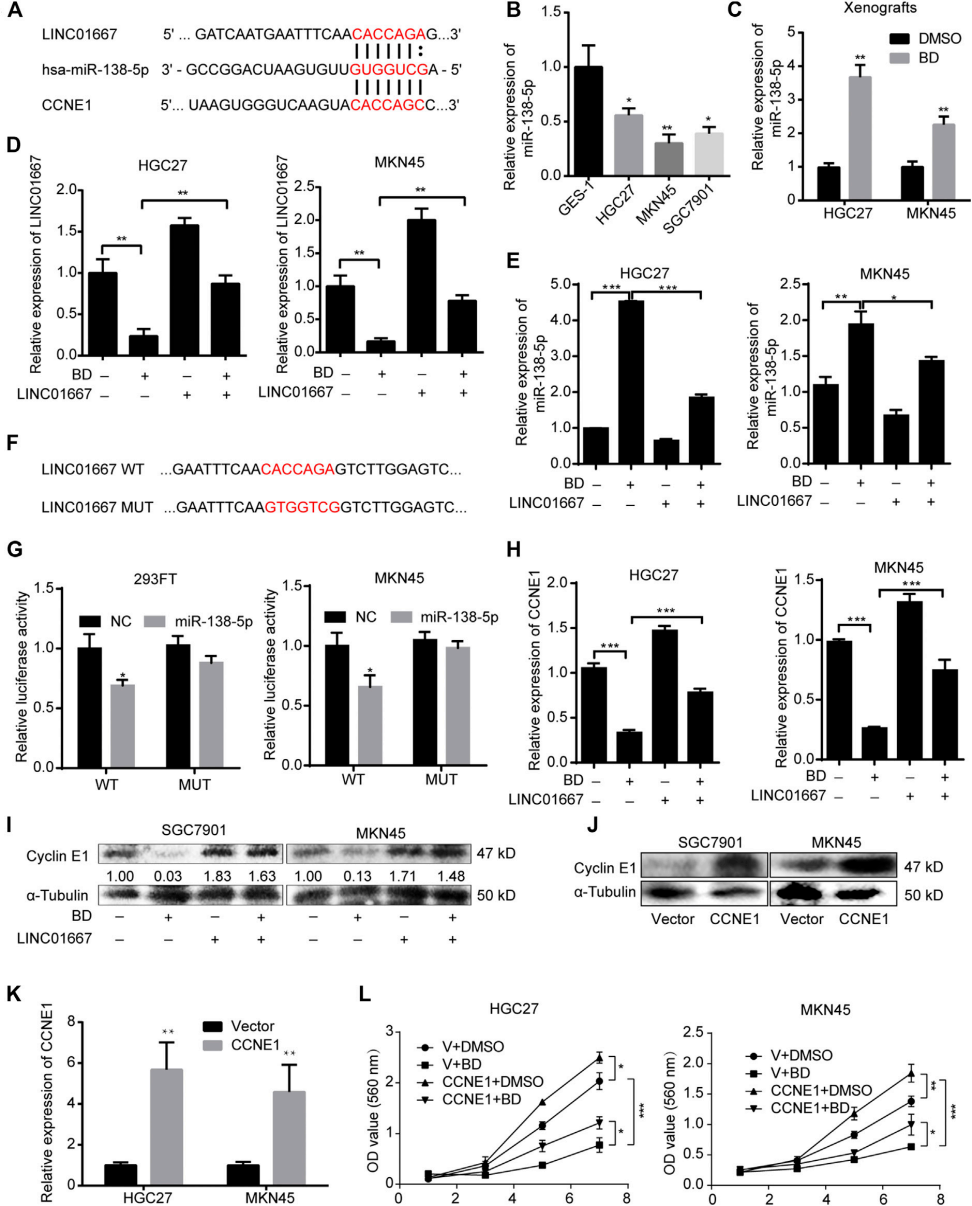
LINC01667 sponges microRNA-138-5p (miR-138-5p) and regulates Cyclin E1 expression. **(A)** The schematic diagram of the binding sites between miR-138-5p and LINC01667 or Cyclin E1 predicted by the TargetScanHuman 7.0. **(B)** The mRNA expression levels of miR-138-5p in GES-1 and gastric cancer cell lines were detected by quantity real-time PCR (qRT-PCR) experiment. **(C)** The mRNA expression levels of miR-138-5p in HGC27 and MKN45 xenografts were detected by qRT-PCR experiment **(D,E)** qRT-PCR assays were performed to evaluate the expression of LINC01667 and miR-138-5p in LINC01667-overexpressed HGC27 and MKN45 cells after treating with bruceine D (BD) at concentrations of 0.4 and 1.2 μM for 48 h, respectively. **(F)** The schematic diagram of LINC01667 wild-type and mutant **(G)** The relative luciferase activity detected by the dual-luciferase reporter assay. **(H)** Cyclin E1 mRNA expression of LINC01667 overexpressing HGC27 and MKN45 cells after treating with BD at concentrations of 0.4 and 1.2 μM for 48 h, respectively. **(I)** The expression levels of Cyclin E1 were determined using the Western blot assay in HGC27 and MKN45 cells after treating with BD at concentrations of 0.4 and 1.2 μM for 48 h, respectively. α-Tubulin was used as control. The gray ratio of Cyclin E1/α-Tubulin was calculated. **(J)** The expression levels of Cyclin E1 were determined using Western blot assay in HGC27 and MKN45 cells after CCNE1 overexpression. **(K)** The relative mRNA expression levels of Cyclin E1 were determined using qRT-PCR in HGC27 and MKN45 cells after CCNE1 overexpression. **(L)** Cell viability was detected by using the MTT assay in HGC27 and MKN45 cells after CCNE1 overexpression and BD treatment. All data were used as mean ± SD, significant difference was tested by the two tailed and unpaired student’s t-test. Error bars, *p < 0.05, **p < 0.01, and ***p < 0.001.

Results above suggested that overexpression of LINC01667 could restore the inhibition of BD on the proliferation of gastric cancer cells. Next, we explored the potential molecular mechanism of this phenomenon. A lncRNA may have many modes of actions via interacting with both proteins, nucleic acids and even metabolites, but the role of sponging microRNAs is the most well studied functions. Therefore, we tried to analyze whether LINC01667-regulated miRNAs also have the potential to regulate cell cycle and cell proliferation in human beings. Interstingly, the binding sites between miR-138-5p and LINC01667 or Cyclin E1 can be predicted by the TargetScanHuman 7.0 (Figure 6A). Importantly, from the database of TargetScanHuman 7.0, the binding site of CACCAGC in the 3′UTR of human are conserved in chimp, rhesus, squirrel, mouse, rat, rabbit, pig, cow, cat, dog, brown bat, elephant and lizard and microRNA-138-5p is the only conserved microRNA that targeted CCNE1 (Supplementary Figure S2; Supplementary Table S1).

As expected, miR-138-5p is downregulated in gastric cancer cell lines, compared with that of the GES-1 cells (Figure 6B). Besides, miR-138-5p was also significantly upregulated after BD treatment in HGC27 and MKN45 xenografts (Figure 6C). To further confirm the relationship between LINC01667 and miR-138-5p, LINC01667 was overexpressed (Figure 6D) and the results revealed that the expression of miR-138-5p was significantly upregulated in gastric cancer cells treated with BD, while overexpression of LINC01667 could inhibit the expression of miR-138-5p (Figure 6E). In order to verify the binding between LINC01667 and miR-138-5p, we constructed the luciferase reporter gene plasmid of LINC01667 wild-type or mutant-type (Figure 6F). Dual-luciferase reporter assays showed that miR-138-5p mimics considerably reduced the luciferase activity of LINC01667-WT, compared with LINC01667-MUT group (Figure 6G).

Since we have showed that Cyclin E1, an important cell cycle regulator, was significantly decreased after BD treatment, we thought that Cyclin E1, one of the potential target genes of miR-138-5p, might be the probable mechanism for regulating cell proliferation in gastric cancer. The results revealed that the expression of Cyclin E1 was remarkably decreased in both mRNA and protein levels after BD treatment in gastric cancer cells, but was recovered in some extent after LINC01667 restoration (Figures 6H,I). To confirm that Cyclin E1 is the downstream target of BD, we overexpressed it (Figures 6J,K) and the results showed that CCNE1 overexpression could rescued the inhibition of cell proliferation induced by BD treatment (Figure 6L).

These results confirm that LINC01667 could sponge miR-138-5p, thereby promoting the expression of Cyclin E1.

### Bruceine D Enhances the Chemosensitivity of Gastric Cancer Cells to Doxorubicin

**FIGURE 7:**
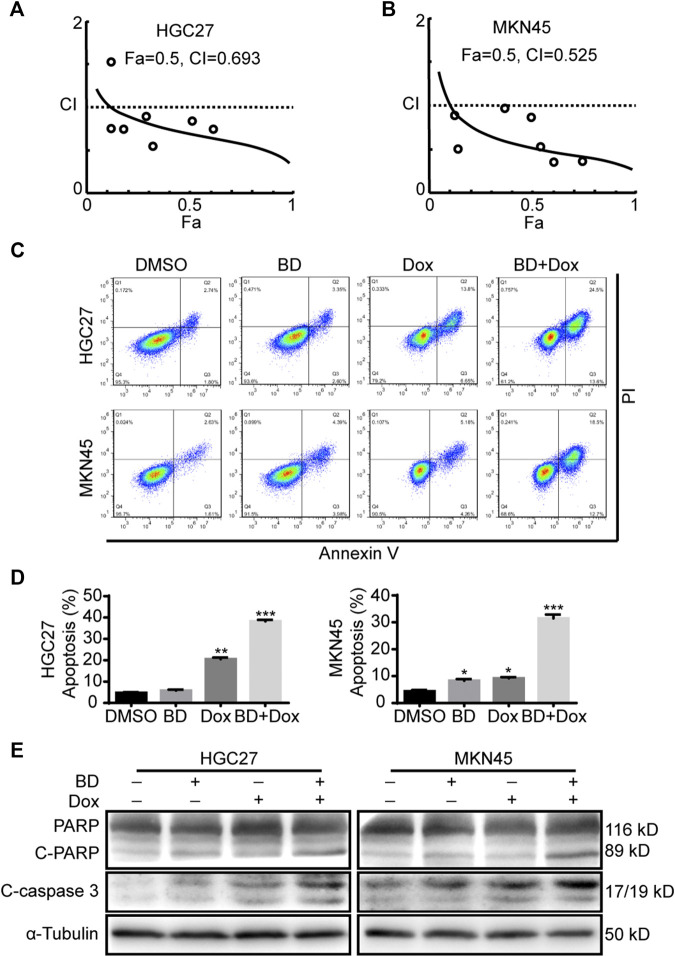
Bruceine D (BD) enhances the chemosensitivity of gastric cancer cells to doxorubicin. **(A,B)** The combined index of BD and doxorubicin were calculated using the CompuSyn software in HGC27 **(A)** and MKN45 **(B)** cells. Fa = 0.5 and combination index (CI) < 1 represents synergy. **(C,D)** The apoptosis of cells was detected by flow cytometry. HGC27 and MKN45 cells were treated with BD and doxorubicin alone or in combination for 48 h, respectively. Dimethyl sulfoxide (DMSO) was used as control. Apoptotic rate of HGC27 and MKN45 cells was quantified. **(E)** The expression of apoptotic proteins, cleaved-PARP and cleaved-caspase3 in cells treated with BD and doxorubicin alone or in combination for 48 h, respectively. α-Tubulin was used as internal reference. All data were used as mean ± SD, significant difference was tested by the two tailed and unpaired student’s t-test. Error bars, *p < 0.05, **p < 0.01, and ***p < 0.001.

Next, we explored whether BD can induce apoptosis of gastric cancer cells. Flow cytometry analysis showed that BD did not induce apoptosis of gastric cancer cells (Figure 7C). Doxorubicin is a commonly chemotherapeutic drug in the treatment of gastric cancer. Therefore, we evaluated whether BD can enhance the chemosensitivity of gastric cancer cells to doxorubicin. Firstly, we used CompuSyn software to judge the combination index of BD and doxorubicin (Figures 7A,B). HGC27 cells were treated with BD (0.4 μM) and doxorubicin (0.4 μM) alone or in combination for 48 h, and MKN45 cells were treated with BD (1.2 μM) and doxorubicin (0.4 μM) alone or in combination for 48 h. The cells were collected for flow cytometry analysis. The results showed that BD significantly increased the cell apoptosis induced by doxorubicin (Figures 7C,D). Western blot analysis confirmed this discovery, and the results showed that the expression of cleaved fragments of PARP and caspase-3 were significantly increased after combination treatment of BD and doxorubicin (Figure 7E). These results suggest that BD can enhance the chemosensitivity of gastric cancer cells to doxorubicin.

## Discussion

Although the incidence has declined over the past few decades, gastric cancer remains as the third leading cause of cancer-related death (Ferlay et al., 2010; Thrift and El-Serag, 2019). Patients with advanced or recurrent gastric cancer have a low survival rate due to drug resistance to chemotherapy (Hu et al., 2015; Chang et al., 2019). Therefore, screening or developing effective therapeutic drugs is one of keys to the treatment of gastric cancer. At present, the treatment of diseases with traditional Chinese medicine has been shown great potential. BD is a quassinoid isolated from the seeds of *Brucea javanica*, which has been used as a traditional Chinese herb to treat a variety of diseases (Zhang et al., 1980; Shen et al., 2008). Recent studies showed that BD had anti-cancer effect in multiple tumor types, including non-small cell lung cancer (Tan et al., 2019), leukemia (Zhang et al., 2016), osteosarcoma (Wang et al., 2019), hepatocellular carcinoma (Cheng et al., 2017) and pancreatic adenocarcinoma (Lau et al., 2009; Yang et al., 2012). However, as far as we know, the effect of BD in gastric cancer still remains unclear.

In this study, we evaluated the anti-tumor activity of BD in gastric cancer. First of all, we demonstrated that BD significantly inhibited tumor growth *in vivo* and *in vitro*. MTT and BrdU assays indicated that BD inhibited the proliferation of gastric cancer cells in a dose-dependent manner. Soft agar assay showed that the colony size became lesser and smaller after BD treatment. Xenograft experiment showed that the tumors formed in nude mice were slower and smaller, and the weight and size of the tumor were significantly reduced after BD treatment. These results suggest that BD can inhibit the growth of gastric cancer cells *in vitro* and *in vivo*.

In recent years, lncRNAs has become a hot spot in tumor research. LncRNAs could be used as a key regulator of epigenetic regulation, transcription and translation to participate in a variety of physiological and pathological processes (Geisler and Coller, 2013; Cech and Steitz, 2014). In this experiment, transcriptome analysis showed that the expression of LINC01667 decreased significantly after BD treatment. LINC01667 is hardly expressed in gastric tissue, but LINC01667 is highly expressed in gastric cancer cells, which suggested that LINC01667 might play an oncogenic role in gastric cancer. MTT and BrdU assays further proved that LINC01667 could restore the inhibition of cell proliferation induced by BD treatment.

Recently, competitive endogenous RNA model has been proved to play a key role in tumorigenesis. In this model, lncRNA can influence other mRNA or lncRNA transcripts by competitively binding to miRNA response element (MRE) to affect post-transcriptional regulation (Cesana et al., 2011; Salmena et al., 2011). In order to explore the potential mechanism, we found that miR-138-5p targeted the mRNA of LINC01667 or Cyclin E1 through the TargetScanHuman 7.0 database. According to previous research reports, miR-138-5p plays an important role in cancer progress. MiR-138 can influence the progression of gastric cancer by regulating EGFR (Wang et al., 2018). LncRNA H19 sponges miR-138-5p, which directly targets SIRT1, and then affects progression of cervical cancer cells (Ou et al., 2018). Long non-coding RNA MCM3AP-AS1 promotes growth and migration through modulating FOXK1 by sponging miR-138-5p in pancreatic cancer (Yang M. et al., 2019). According to the target binding sites, we constructed LINC01667-WT and LINC01667-MUT double luciferase vectors. Next, dual-luciferase reporter assays showed that miR-138-5p could significantly reduce the luciferase activity of LINC01667-WT, compared with LINC01667-MUT. These experimental results showed that LINC01667 could target miR-138-5p.

Unlimited cell proliferation is one of the most distinctive features of tumor. Cell cycle is closely related to cell proliferation. Some studies have confirmed that the catalytic activity of CDK is regulated by Cyclins and CDK inhibitor (CKI), and the close cooperation between them is very important for the normal advancement of the cell cycle. Based on the above results from the cell cycle and Western blotting assays, we found that BD arrested gastric cancer cells in S phase by down-regulating Cyclin E1, Cyclin E2 and CDK2. CCNE1 (cyclin E1) mRNAs are significantly higher in GC tissues than in normal tissues in both Oncomine and The Cancer Genome Atlas (TCGA) datasets (Zhang et al., 2018) and this was also confirmed by the multiplex ligation-dependent probe amplification and fluorescence *in situ* hybridization (Ooi et al., 2017). Besides, it was also revealed that upregulated Cyclin E1 is highly associated gastric cancer development (Gu et al., 2010) and liver metastasis (Chang et al., 2009; Kim et al., 2019). In our study, qRT-PCR experiments showed that BD treatment could significantly reduce the expression of Cyclin E1, while overexpression of LINC01667 could restore the expression of Cyclin E1 to some extent. Besides, overexpression of CCNE1 also recovered cell proliferation inhibition induced by BD treatment to some extent. These results indicated that BD could inhibit the proliferation of gastric cancer cells through LINC01667/miR-138-5p/Cyclin E1 pathway. However, the binding activity and the association between miR-138-5p and CCNE1 mRNA should be further confirmed.

Cyclin E2 are also shown to be upregulated at the early stage of gastric cancer (Kumari et al., 2016). CDK2 may also contributes to gastric progression via promoting gluycolysis (Tang et al., 2018). In our study, these two factors were also significantly inhibited by BD treatment, indicating Cyclin E2 and CDK2 might also be the potential target genes. However, we did not found their connections with LINC01667. They might be affected by BD through other unknown mechanisms. Besides, whether there were other mechanisms except for LINC01667/miR-138-3p/CCNE1 needs to be explored in the future research.

Drug resistance to therapeutic drugs is one of the main reasons for the low efficacy of cancer treatment. Some studies have shown that the extraction of traditional Chinese medicine can improve the drug sensitivity of cancer cells. Such as, scutellarin enhanced the sensitivity of prostate cancer cells to cisplatin (Gao et al., 2017). Corilagin sensitized epithelial ovarian cancer cells to paclitaxel and carboplatin treatment by inhibiting snail-glycolysis pathways (Jia et al., 2017). Triptolide enhanced the sensitivity of breast cancer cells to doxorubicin (Deng et al., 2018). Doxorubicin is one of the commonly used chemotherapeutic drugs in the treatment of gastric cancer (Murad et al., 1993). Therefore, we evaluated whether BD could enhance the chemosensitivity of gastric cancer cells to doxorubicin. We found that the apoptosis of cells treated with BD and doxorubicin was significantly higher than that treated with BD or doxorubicin alone. At the same time, the Western blot assay were carried out. The results showed that the expression of apoptotic proteins PARP and caspase-3 were significantly increased after combination treatment with BD and doxorubicin. Therefore, these results confirm that BD can enhance the chemosensitivity of gastric cancer cells to doxorubicin.

**FIGURE 8:**
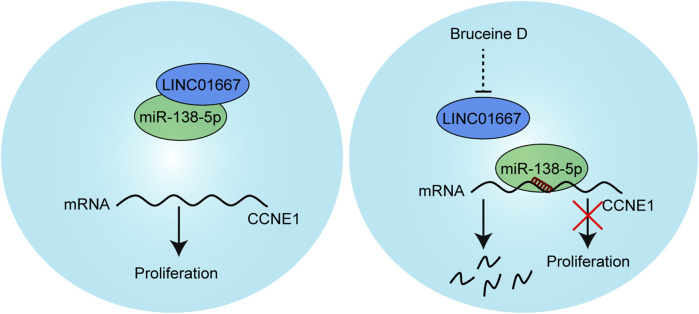
Model of action for the BD/LINC01667/microRNA-138-5p (miR-138-5p)/Cyclin E1 regulatory axis in the modulation of cell proliferation in gastric cancer cells. Briefly, under normal conditions, LINC01667 can competitively adsorb miR-138-5p. When treated with bruceine D (BD), the expression of LINC01667 was down-regulated, and miR-138-5p combined with CCNE1 mRNA, which inhibited the expression of Cyclin E1 and inhibited the proliferation of cells.

In summary, we identify that BD inhibits the proliferation of gastric cancer cells through the LINC01667/miR-138-5p/Cyclin E1 pathway (Figure 8). More importantly, BD can enhance the chemosensitivity of gastric cancer cells to doxorubicin. Our results provide a new perspective for the molecular pathogenesis of gastric cancer and initially suggest that BD may be a promising drug for the treatment of gastric cancer.

## Data Availability Statement

The original contributions presented in the study are included in the article/Supplementary Material, further inquiries can be directed to the corresponding authors.

## Ethics Statement

The animal study was reviewed and approved by Animal Ethics Committee of Southwest University.

## Author Contributions

LL, ZD, LY, and HC study design; LL, PS, LT, JX, PH, and ZW acquisition of data; LL and ZD analysis and interpretation of data; LL and ZD drafting of the manuscript; LL and ZD statistical analysis; LY and HC funding and study supervision. All authors read and approved the final manuscript.

## Funding

We are grateful for funding support from the National Natural Science Foundation of China (Nos. 31672496, 81201551, 81902664, 81872071, and 81672502), the Fundamental Research Funds for the Central Universities (Nos. XDJK2020B006 and SWU120009), the Research and Innovation Project of Graduate Students in Chongqing (No. CYS19136), the Eyas Program of the Youth Innovative Talents Cultivation in Chongqing (No. CY200237), the National Key Research and Development Program of China (Nos. 2017YFC1308600 and 2016YFC1302204), and the Natural Science Foundation of Chongqing (No. cstc2019jcyj-zdxmX0033).

## Conflict of Interest

The authors declare that the research was conducted in the absence of any commercial or financial relationships that could be construed as a potential conflict of interest.
