# Supplementary material for: Bruceine D inhibits Cell Proliferation Through Downregulating LINC01667/MicroRNA-138-5p/Cyclin E1 Axis in Gastric Cancer
Source: Front Pharmacol. 2020 Nov 24;11:584960. doi: 10.3389/fphar.2020.584960 (PMC7774499; doi:10.3389/fphar.2020.584960)
Supplement: Supplementary file 1 [file datasheet1.docx]

**Supplemental Data**


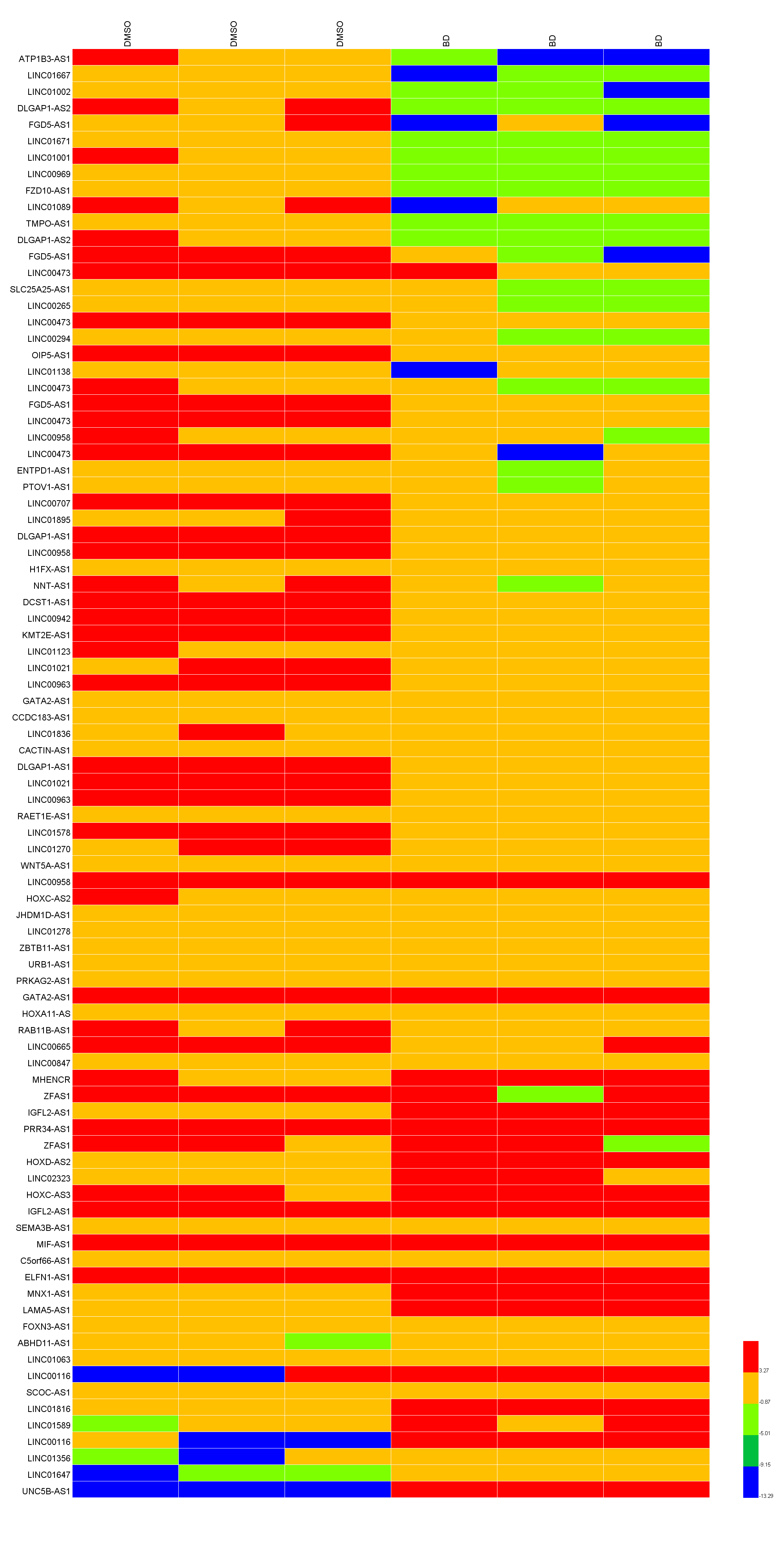


**Supplemental Figure 1.** Heatmap clustering of the transcriptome data regarding to the different expressions of long non-coding RNAs in MKN45 gastric cancer cell lines after treating with 1.2 μM BD or isometric DMSO for 48 h. Only lncRNAs with qValue<0.05 and FoldChange>2 were shown in this heatmap. Heml 1.0: Heatmap Illustrator was used for drawing the heatmap according to the log(MeanTPM) of each transcript. Please note that some lncRNAs such as LINC00116, DLGAP1-AS1 and DLGAP1-AS1 have more than one transcripts. The rank of these lncRNAs are listed according to the mean fold change of the MeanTPM in DMSO/BD.


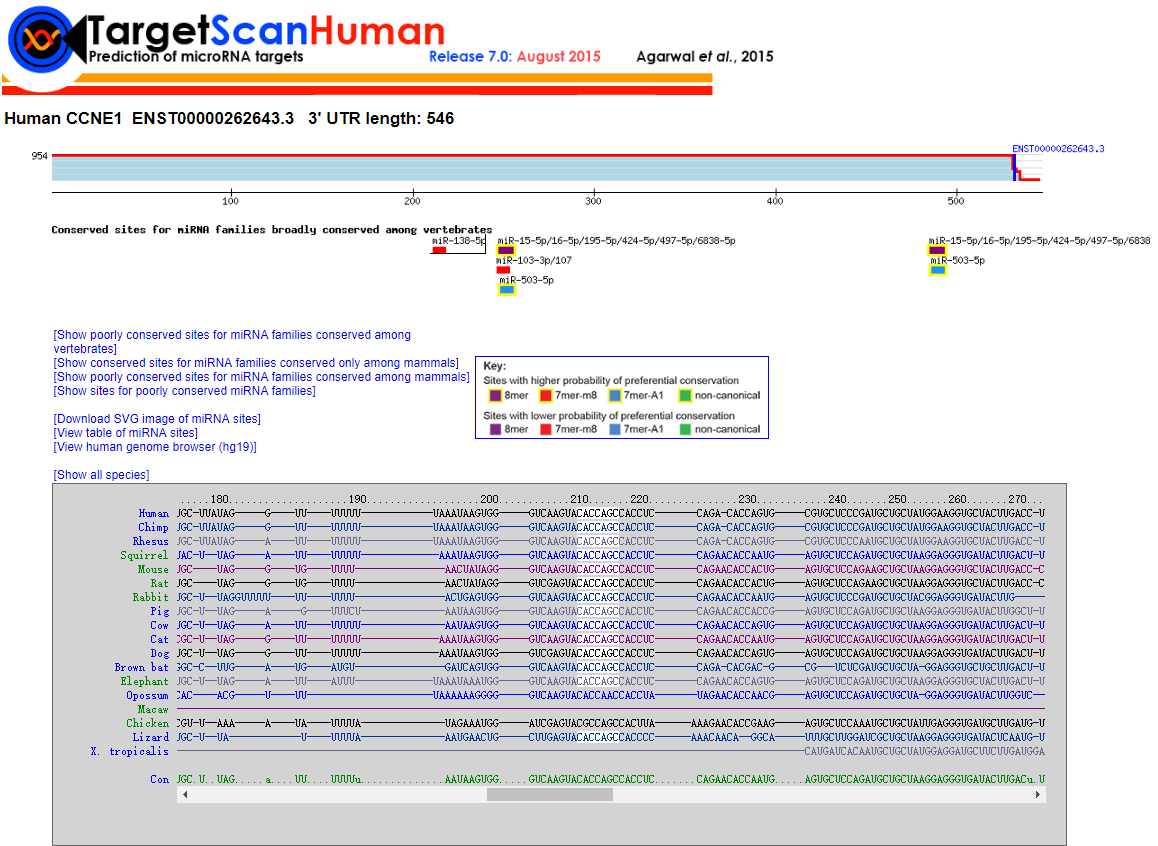


**Supplemental Figure 2.** Predicted targeted microRNAs in the 3’UTR of CCNE1 by TargetScan

**Supplemental Table 1.** Predicted conserved targeted microRNAs in the 3’UTR of CCNE1 by TargetScan

|  | **Predicted consequential pairing of target region (top) and miRNA (bottom)** | **Site type** | **Context++ score** | **Context++ score percentile** | **Weighted context++ score** | **Conserved branch length** | **P_CT_** |
| --- | --- | --- | --- | --- | --- | --- | --- |
| Position 211-217 of CCNE1 3' UTR | 5' ...UAAGUGGGUCAAGUA**CACCAGC**C. | 7mer-m8 | -0.36 | 95 | -0.36 | 3.881 | 0.55 |
| hsa-miR-138-5p | 3' GCCGGACUAAGUGUU**GUGGUCG**A |  |  |  |  |  |  |
